# Maternal Stress, Anxiety, Well-Being, and Sleep Quality in Pregnant Women throughout Gestation

**DOI:** 10.3390/jcm12237333

**Published:** 2023-11-26

**Authors:** Rosalia Pascal, Irene Casas, Mariona Genero, Ayako Nakaki, Lina Youssef, Marta Larroya, Leticia Benitez, Yvan Gomez, Anabel Martinez-Aran, Ivette Morilla, Teresa M. Oller-Guzmán, Andrés Martín-Asuero, Eduard Vieta, Fàtima Crispi, Eduard Gratacos, María Dolores Gomez-Roig, Francesca Crovetto

**Affiliations:** 1 BCNatal (Hospital Sant Joan de Déu and Hospital Clínic), University of Barcelona, Passeig Sant Joan de Déu, 2, 08959 Esplugues de Llobregat, Spain; rosalia.pascal@sjd.es (R.P.); irene.casas@sjd.es (I.C.); mariona.genero@sjd.es (M.G.); lyoussef@recerca.clinic.cat (L.Y.); larroya@clinic.cat (M.L.); lbenitez@clinic.cat (L.B.); yvan.gomez@chuv.ch (Y.G.); fcrispi@clinic.cat (F.C.); egratacos@ub.edu (E.G.); lola.gomezroig@sjd.es (M.D.G.-R.); 2 Primary Care Interventions to Prevent Maternal and Child Chronic Diseases of Perinatal and Development Origin, RD21/0012/0003, Instituto de Salud Carlos III, 28040 Barcelona, Spain; 3 Institut de Recerca Sant Joan de Déu (IRSJD), 08950 Barcelona, Spain; 4 Institut D’investigacions Biomèdiques August Pi Sunyer (IDIBAPS), 08036 Barcelona, Spain; 5 Josep Carreras Leukaemia Research Institute, Hospital Clinic/University of Barcelona Campus, 08036 Barcelona, Spain; 6 Department of Psychiatry and Psychology, Hospital Clinic, Neuroscience Insititute, IDIBAPS, University of Barcelona CIBERSAM, 08035 Barcelona, Spain; amartiar@clinic.cat (A.M.-A.); imorilla@clinic.cat (I.M.); evieta@clinic.cat (E.V.); 7 Instituto esMindfulness, 08015 Barcelona, Spain; m.teresa@esmindfulness.com (T.M.O.-G.); andres@esmindfulness.com (A.M.-A.); 8 Center for Biomedical Network Research on Rare Diseases, 28029 Madrid, Spain

**Keywords:** mental stress, anxiety, well-being, sleep quality, pregnancy

## Abstract

Background: Maternal stress, anxiety, well-being, and sleep quality during pregnancy have been described as influencing factors during pregnancy. Aim: We aimed to describe maternal stress, anxiety, well-being, and sleep quality in pregnant women throughout gestation and their related factors. Methods: A prospective study including pregnant women attending BCNatal, in Barcelona, Spain (*n* = 630). Maternal stress and anxiety were assessed by the Perceived Stress Scale (PSS) and State-Trait Anxiety Inventory (STAI)-validated questionnaires. Maternal well-being was assessed using the World Health Organization Well-Being Index Questionnaire (WHO-5), and sleep quality was assessed using the Pittsburgh Sleep Quality Index Questionnaire (PSQI). All questionnaires were obtained twice during the second and third trimester of pregnancy. A multivariate analysis was conducted to assess factors related to higher maternal stress and anxiety and worse well-being and sleep quality. Results: High levels of maternal stress were reported in 23.1% of participants at the end of pregnancy, with maternal age <40 years (OR 2.02; 95% CI 1.08–3.81, *p* = 0.03), non-white ethnicity (OR 2.09; 95% CI 1.19–4.02, *p* = 0.01), and non-university studies (OR 1.86; 95% CI 1.08–3.19, *p* = 0.02) being the parameters mostly associated with it. A total of 20.7% of women had high levels of anxiety in the third trimester and the presence of psychiatric disorders (OR 3.62; 95% CI 1.34–9.78, *p* = 0.01) and non-university studies (OR 1.70; 95% CI 1.11–2.59, *p* = 0.01) provided a significant contribution to high anxiety at multivariate analysis. Poor maternal well-being was observed in 26.5% of women and a significant contribution was provided by the presence of psychiatric disorders (OR 2.96; 95% CI 1.07–8.25, *p* = 0.04) and non-university studies (OR 1.74; 95% CI 1.10–2.74, *p* = 0.02). Finally, less sleep quality was observed at the end of pregnancy (*p* < 0.001), with 81.1% of women reporting poor sleep quality. Conclusion: Maternal stress and anxiety, compromised maternal well-being, and sleep quality disturbances are prevalent throughout pregnancy. Anxiety and compromised sleep quality may increase over gestation. The screening of these conditions at different stages of pregnancy and awareness of the associated risk factors can help to identify women at potential risk.

## 1. Introduction

According to the World Health Organization (WHO), health is a “state of complete physical, mental, and social well-being, and not merely the absence of disease or infirmity”. Therefore, mental health, defined by the WHO as a “state of mental well-being that enables people to cope with the stresses of life, realize their abilities, learn well and work well, and contribute to their community”, is as fundamental as physical health in the achievement of positive overall wellness in an individual [1].

Stress, anxiety, compromised mental well-being, and sleep quality are fundamental and interconnected aspects of mental health. They can impact each other and together contribute to a general state of emotional and mental wellness. Mental stress can be medically understood as the ‘individual’s perception of a stimulus as overwhelming’ which results in a response and a transformed state [2]. Anxiety is defined by the American Psychological Association as “an emotion characterized by feelings of tension, worried thoughts, and physical changes like increased blood pressure.” Both stress and anxiety are emotional responses. Stress is usually precipitated by an external factor, whereas anxiety is defined by the persistence of excessive worries even in the absence of a stressor. Well-being is broadly defined as ‘the quality and state of a person’s life’ [3] and consists of two components: feeling healthy and relatively robust and being able to carry out one’s job and other tasks satisfactorily [4]. Finally, sleep quality is defined as an individual’s level of satisfaction with all aspects of the sleep experience [5]. Sleep quality is highly dependent on the person’s general well-being. 

Maternal mental stress, anxiety, compromised well-being, and sleep quality have been associated with several adverse pregnancy outcomes such as preterm birth (PTB) [6,7,8,9,10,11,12], low birthweight (LBW) [7,13,14,15], gestational diabetes (GD) [16,17], labor complications [12,18,19,20,21], or hypertension and preeclampsia (PE) [22,23]. Moreover, maternal stress has been demonstrated to be a prenatal programming factor that affects the fetal neurodevelopment [24] and could compromise the socioemotional competencies in childhood that are the foundation for future well-being [24].

Mental stress, anxiety, compromised well-being, and sleep disturbances are common during pregnancy. Around 20% of pregnant women could experience excessive concern regarding future events in pregnancy under normal circumstances [4]. Up to 70% of pregnant women report symptoms of stress and anxiety during pregnancy, with between 10% and 16% of them fulfilling the criteria for a major depressive disorder [25,26]. While the real prevalence of antenatal psychosocial stress is still unclear [27], in a 2003 study, Rondó et al. found high stress in 22–25% of pregnant women during the three trimesters of pregnancy [7]. In a meta-analysis of 102 studies involving 221,974 women, Dennis et al. found that the prevalence rate for self-reported anxiety symptoms in the first trimester was 18.2% and 24.6% in the third trimester [28]. These percentages decreased when employing diagnostic interviews: the prevalence rate for any anxiety disorder during the first trimester was 18% and 15% in the final two trimesters of pregnancy [28]. However, we can speculate that the symptoms of depression can overlap with some normal feelings during pregnancy, which could explain such high percentages and the disparity found among studies [26]. There is no clear evidence of the prevalence of compromised well-being during pregnancy. A highly variable prevalence of poor sleep quality in pregnant women has also been reported, ranging from 17% to 76% [29]. This disparity could be due to dissimilar sample compositions and different methods and timings of assessments [30]. Moreover, some authors have even postulated the possibility that the previously validated cut-off values for sleep questionnaires in the general population may not be valid in pregnancy, thus requiring a higher score [30].

Different risk factors for antenatal mood disorders have been postulated in the previously published literature. Sociodemographic variables such as age have been considered in multiple studies with inconsistent findings among them [31,32]. Other sociodemographic variables considered in the previous literature are maternal socioeconomic status and educational level: in a 2010 systematic review, Lancaster et al. found a small association between low educational level and depression symptoms that could not be demonstrated in the multivariate analyses [33]. Later, Biaggi et al. found low maternal educational level to be associated with anxiety and depressive symptoms [32]. As for ethnicity, socioeconomic status, employment, an unfavorable socioeconomic situation, unemployment, and belonging to a minority ethnic group are associated with depression in several studies [31,32,34] but inconsistent results are described in others [32,33]. On the other hand, other factors such as smoking, alcohol intake, and drug abuse showed inconsistent findings in their association with depression and sleep quality [29,32,33,34]. A personal medical history of anxiety and depression has strongly been associated with perinatal depression [31,32,33,34]. Other studies suggest an association between previous obstetric history, like previous abortions or pregnancy complications, with depressive symptoms and poor sleep quality [29,31,32] but also with inconsistent findings [33]. A complex multifactorial origin for the etiology of these conditions could be a possible explanation for such different results reported in the literature [33].

Despite the high prevalence of these antenatal negative affective states and their impact on pregnancy, it is still unclear if they worsened during pregnancy and what the potential risk factors for these conditions are during pregnancy.

The aim of this study was to determine maternal stress, anxiety, well-being, and sleep quality across different stages of pregnancy and to identify related risk factors. 

## 2. Materials and Methods

### 2.1. Study Design and Participants

A prospective study was carried out at BCNatal (Hospital Clinic and Hospital Sant Joan de Déu), a large referral center for maternal-fetal and neonatal medicine in Barcelona, Spain. Inclusion criteria were pregnant women with a singleton fetus who attended our center for their second trimester scan (19–23 weeks of gestation), and who were able to respond to maternal stress, anxiety, well-being, and sleep quality validated questionnaires. The exclusion criteria for the study are as follows: maternal mental retardation or other mental or psychiatric disorders that raise doubts regarding the patient’s real willingness to participate in the study and the impossibility of completing questionnaires or other procedures in the study, congenital infections, fetal anomalies including chromosomal abnormalities or structural malformations detected by ultrasound prenatally, and neonatal abnormalities diagnosed after birth. The study was approved by the hospital ethical committee (HCB-2016-0830 and HCB/2020/0209) and written informed consent was obtained from all participants.

### 2.2. Study Aims

The main aim of the study was to evaluate maternal stress, anxiety, well-being, and sleep quality at two moments during pregnancy, assessed using four different validated questionnaires: the Perceived Stress Scale (PSS) [35] and State-Trait Anxiety Inventory (STAI) [36] for maternal stress and anxiety, respectively, the World Health Organization Well-Being Index Questionnaire (WHO-5 Index) for maternal well-being [37], and the Pittsburgh Sleep Quality Index (PSQI) [38] for sleep quality.

The secondary aim was to evaluate maternal and pregnancy factors acting as potential risk factors for increased maternal stress and anxiety, poorer maternal well-being status, and poorer sleep quality during gestation.

### 2.3. Data Collection

All questionnaires were completed twice during pregnancy: at recruitment of the study population in their second trimester of pregnancy (19–23 weeks of gestation) and again at the end of the third trimester of pregnancy (34–36 weeks of gestation).

The Perceived Stress Scale was designed to measure “the degree to which individuals appraise situations in their lives as stressful” [35]. It is a brief scale, consisting of only 14 items evaluating stress within the last 8 weeks. PSS scores are obtained by reversing responses to the 4 positively stated items (items 4, 5, 7, and 8) and then adding across all scale items. It is not a diagnostic instrument; therefore, there are no cut-offs for classification of the stress, but it gives a comparison instrument between people [39]. The higher stress group in this cohort was considered the 75th percentile at the first evaluation (19–23 weeks of gestation). 

The STAI questionnaire consists of two subscales: the State Anxiety Scale (STAI-S), which evaluates the current state of anxiety, and the Trait Anxiety Scale (STAI-T), which evaluates individual aspects of “anxiety proneness”. The STAI has 40 items, 20 items allocated to each of the S-State and T-Trait subscales. The range of scores for each subtest is 20–80, the higher indicating greater anxiety [40,41]. The higher stress group in this cohort was considered the 75th percentile at the first evaluation (19–23 weeks of gestation). 

The WHO-5 consists of a five-item scale and it is used to rate quality of life and psychological well-being, according to the participant’s feelings within the last 15 days. The raw score ranges from 0 to 25: 0 representing worst possible and 25 representing best possible quality of life. Following total scores, standardized scores (0–100) are calculated. Women were classified according to their well-being status as with a poor (≤52) or favorable (>52) WHO-5 score [42].

The PSQI assesses sleep quality and disturbances over a monthly interval. It contains 19 self-rated questions which are combined to form 7 component scores: subjective sleep quality, sleep latency, sleep duration, habitual sleep efficiency, sleep disturbances, use of sleeping medication, and daytime dysfunction. Each of these components has a range of 0–3 points (where 0 means no difficulty and 3 indicates severe difficulty). The 7 component scores are added to give a global score, with a range of 0–21 points, 0 indicating no difficulty and 21 indicating severe difficulties in all areas. A global PSQI score greater than 5 defines poor sleep quality [38]. 

Baseline and socioeconomic characteristics, such as maternal age, ethnicity, educational level, or pre-pregnancy body mass index (BMI) were obtained from a structured questionnaire. Medical and obstetric history were obtained from the medical records at recruitment.

### 2.4. Statistical Analysis

For the first aim, the analysis was based on the scores of PSS, STAI-S, STAI-T, WHO-5, and PSQI-validated questionnaires. Continuous variables were assessed for normality using the Shapiro–Wilk’s test. Normally distributed variables were compared using a t-test and expressed as mean and standard deviation (SD). Non-normally distributed variables were compared using the U–Mann–Whitney test and expressed as the median and interquartile range (IQR). Categorical variables were compared using χ^2^ or Fisher’s exact test where appropriate. To study the correlation of the different tests, Pearson correlation analyses were performed. For the secondary outcomes, logistic regression analysis with forward stepwise selection was performed to assess the association between maternal higher stress (>p75) (PSS, STAI-S, STAI-T), poor well-being (≤52 WHO-5), and lower sleep quality (>5 PSQI), with potential maternal risk factors at final evaluation (34–36 weeks of gestation). A multivariate analysis was performed for the variables found to have a significant effect in bivariate analyses. The odds ratio (OR) and a 95% confidence interval (95% CI) were calculated. A *p*-value < 0.05 was considered statistically significant. The analysis was performed using SPSS v26 (New York, NY, USA).

## 3. Results

### 3.1. Study Population

A total of 630 women were recruited in the second trimester at a median [IQR] gestational age of 20 weeks [20,21]). The majority of women (n = 497, 79.3%) were of white ethnicity and with university studies (n = 427, 68%). Baseline characteristics of the study population are shown in Table 1. Regarding their medical history, 2.7% of women (n = 17) had psychiatric disorders requiring therapy, 5.6% (n = 35) had thyroid disorders, and 7.8% (n = 49) had a BMI ≥ 30.

### 3.2. Stress, Anxiety, Well-Being, and Sleep Quality throughout Pregnancy

**Figure 1:**
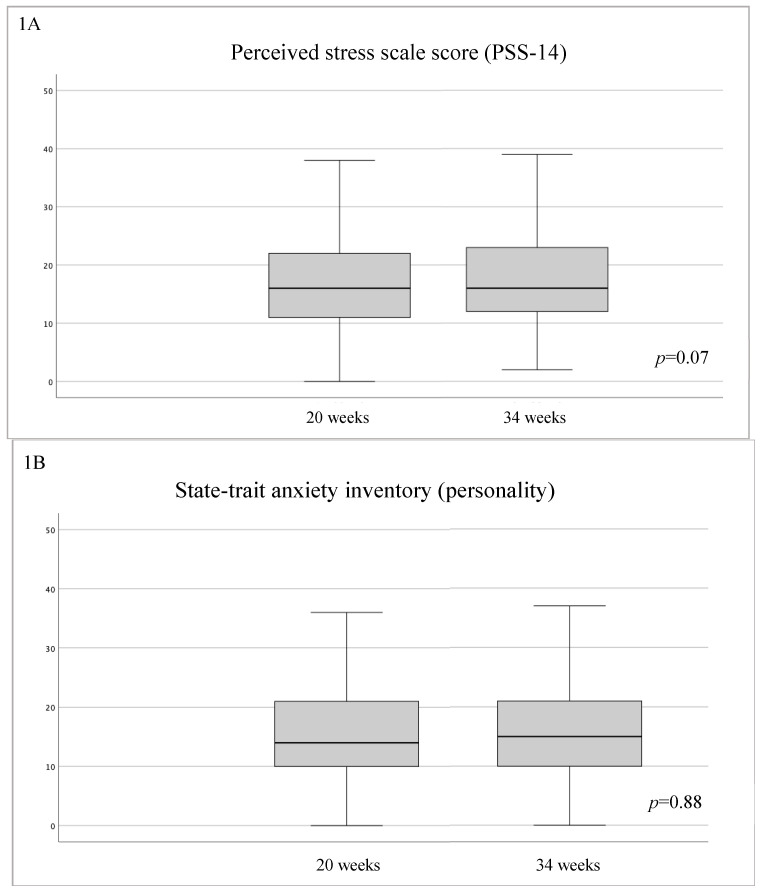

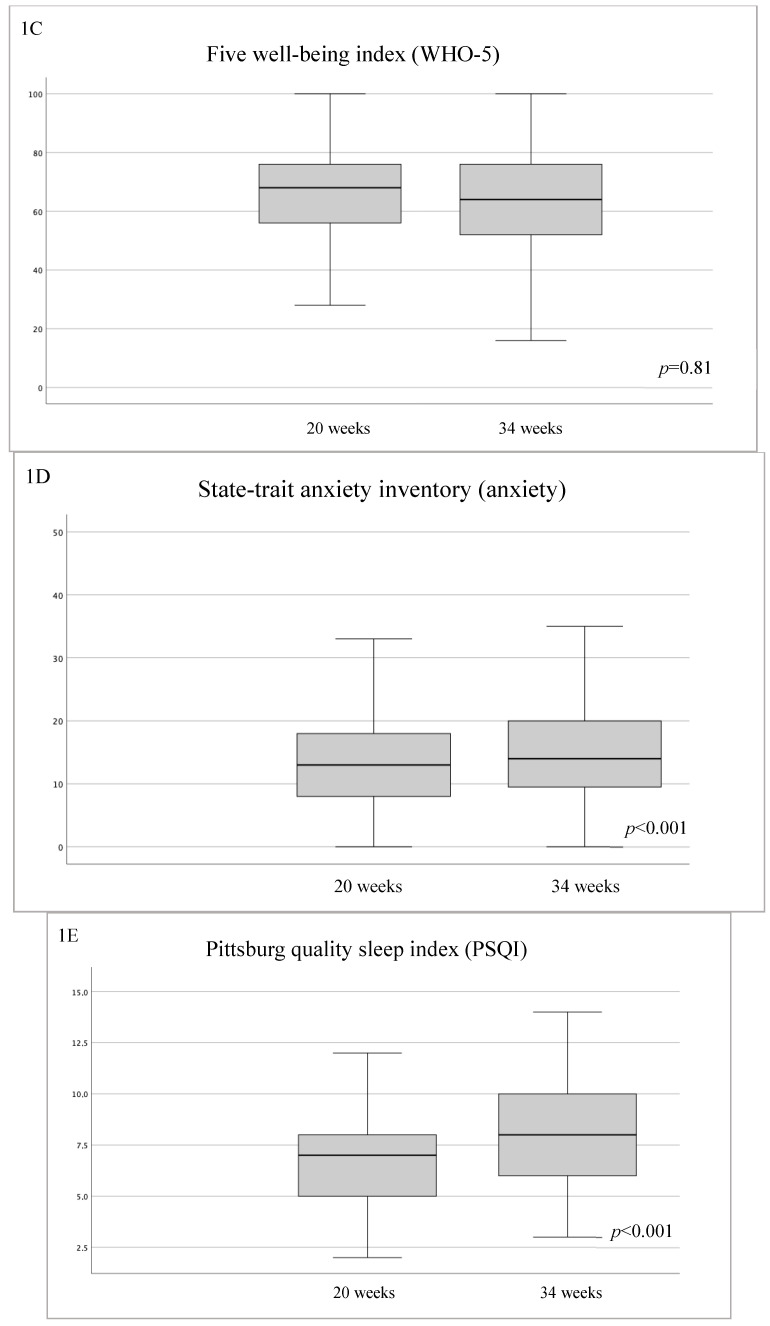
(**A**) Evolution in PSS-14 test score at baseline (20 weeks) and at the end of gestation (34 weeks). The median (IQR) scores of the PSS at second trimester evaluation was 16 (11–22) and it did not change during pregnancy. (**B**) Evolution in STAI-T test score at baseline (20 weeks) and at the end of gestation (34 weeks). No changes during gestation were found. (**C**) Evolution in WHO-5 test score at baseline (20 weeks) and at the end of gestation (34 weeks). No changes during gestation were found. (**D**) Evolution in STAI-S test score at baseline (20 weeks) and at the end of gestation (34 weeks). An increased score during the third trimester was observed. (**E**) Evolution in PSQI test score at baseline (20 weeks) and at the end of gestation (34 weeks). An increased score during the third trimester was observed.

The median [IQR] scores of the PSS at the second trimester evaluation was 16 (11–22), and it did not change during pregnancy, as reported in Table 2 and Figure 1A. No changes during gestation were found for STAI-T and for the well-being evaluation (WHO-5) (see Table 2 and Figure 1B,C). On the contrary, an increasing score during the third trimester was observed for the STAI-S (*p* < 0.001) and PSQI questionnaires (*p* < 0.001) (see Table 2 and Figure 1D,E).

The correlation between the final results of the stress and anxiety tests was calculated by the Pearson correlation coefficient, which showed a significative positive strong correlation between the levels of stress and anxiety (PSS vs. STAI-S, r = 0.72, *p* < 0.001; PSS vs. STAI-T, r = 0.69, *p* < 0.001; STAI-T vs. STAI-S, r = 0.75, *p* < 0.001). A significative negative moderate correlation was observed between WHO-5 and the stress and anxiety tests, highlighting poorer mental well-being in relation to higher levels of anxiety and stress (WHO-5 vs. PSS, r = −0.58, *p* < 0.001; WHO-5 vs. STAI-S, r = −0.63, *p* < 0.001; WHO-5 vs. STAI-T, r = −0.65, *p* < 0.001). Finally, the correlation found between sleep quality and stress, anxiety, and mental well-being was low (PSQI vs. STAI-S, r = 0.31, *p* < 0.001; PSQI vs. STAI-T, r = 0.34, *p* < 0.001; PSQI vs. PSS, r = 0.33, *p* < 0.001; PSQI vs. WHO-5, r = 0.40, *p* < 0.001).

### 3.3. Maternal Stress and Anxiety

High levels of maternal PSS were reported in 115 women (23.1%) at the end of pregnancy. At multivariate analysis, a significant contribution to this condition was provided by maternal age <40 years (OR 2.02; 95% CI 1.08–3.81, *p* = 0.03), non-white ethnicity (OR 2.09; 95% CI 1.19–4.02, *p* = 0.01), and non-university studies (OR 1.86; 95% CI 1.08–3.19, *p* = 0.02). Details are reported in Table 3. 

According to the STAI questionnaire (anxiety, STAI-S), 129 women (20.7%) had high levels of anxiety in the third trimester. In these women, a significant contribution to multivariate analysis was provided by the presence of psychiatric disorders (OR 3.62; 95% CI 1.34–9.78, *p* = 0.01), and non-university studies (OR 1.70; 95% CI 1.11–2.59, *p* = 0.01). Details are reported in Table 4.

According to the STAI-T personality questionnaire, 116 women (23.6%) ended pregnancy with a high anxiety trait level. In the multivariate analysis, a significant contribution to this condition was provided by maternal age <40 years (OR 2.07; 95% CI 1.11–3.88, *p* = 0.02) and preeclampsia in a previous pregnancy (OR 2.9; 95% CI 1.03–8.2, *p* = 0.04). Details are reported in Table 5.

### 3.4. Maternal Well-Being

Poor maternal well-being (WHO-5 score ≤52) was observed in 131 women (26.5%) in the 3rd trimester assessment. Significant contribution to a low maternal well-being was provided by the presence of psychiatric disorders (OR 2.96; 95% CI 1.07–8.25, *p* = 0.04), and non-university studies (OR 1.74; 95% CI 1.10–2.74, *p* = 0.02). Details are reported in Table 6.

### 3.5. Maternal Sleep Quality

Poor maternal sleep quality affected 309 women (81.1%) at 34–36 weeks of gestation. While non-white ethnicity (OR 2.74; 95% CI 1.13–6.61, *p* = 0.03) and obesity (OR 2.01; 95% CI 1.07–3.79, *p* = 0.03) were significant contributors to low maternal sleep quality in the univariate analysis, in the multivariate analysis no significant contributing factors were found, as reported in Table 7.

## 4. Discussion

Our study reveals the potential importance of assessing antenatal negative affective states in a pregnant population. Stress, anxiety, compromised well-being, and sleep disorders have been reported by a significant number of pregnant participants in our cohort. There is a possible underassessment of these conditions by obstetric-care providers in daily clinical practice and our results stress the importance of actively evaluating signs and symptoms of negative affective states and sleep quality throughout gestation. 

Perceived stress and STAI-T did not change throughout pregnancy; however, STAI-S increased in the third trimester of pregnancy. Previously published studies have shown that anxiety and depressive symptoms are not homogeneous during the perinatal period [32,43,44]. Thus, nearly one quarter of participants scored as high stress and anxiety in the third trimester of pregnancy. Such percentages of perceived stress and anxiety highlight the importance of targeting these patients with clinically validated questionnaires in routine pregnancy follow-ups, with the aim of offering support interventions to these patients. Moreover, previous evidence has suggested that pregnancy-related anxiety constitutes a different concept from general anxiety. This fact could be a possible explanation for a limited measurement and assessment of anxiety in pregnancies and could also encourage the need for research in pregnancy-adapted measurement tools [45].

To the best of our knowledge, there are no data regarding the prevalence of compromised well-being in the pregnant population with which to compare our results. However, in a study conducted by Sattler et al. in a group of overweight and obese women in Europe, a prevalence of low well-being of 27% before 20 weeks of pregnancy is reported [46]. Similarly, during the COVID-19 pandemic Mortazavi et al. reported a prevalence of compromised wellbeing of 24.4% pregnant women during gestation [4]. Around 26% of our population had compromised well-being, which is a similar percentage. The WHO-5 questionnaire is considered a good screening questionnaire with high sensitivity and specificity for clinical depression [46]. It has the advantage of being a relatively easy and quick instrument to use in daily clinical practice allowing a first detection of women with a negative affective state who could benefit from a further mental health assessment.

The prevalence of sleep disturbances in our cohort was very high: more than 80% of participants were found to have compromised sleep at 34–36 weeks of gestation. Our prevalence results are higher than expected according to the literature, ranging from 17% to 76% [29]. As suggested in previous studies, this fact could highlight the possibility that the validated cut-off for sleep questionnaires in the general population may not be valid in pregnancies, the latter requiring a higher score [30]. Moreover, we found that the results of sleep quality questionnaires worsened in the third-trimester assessment as compared to the results found in the previous weeks of gestation. The worsening of sleep quality throughout gestation identified in our cohort is in line with previous evidence: according to a meta-analysis of 24 studies, it was found that sleep disturbances tend to increase during pregnancy and clinicians should be aware that complaints of very poor sleep could require intervention [30]. 

Diagnosis and screening of maternal mental health have long been recommended by scientific societies. For instance, the American College of Obstetricians and Gynecologists recommends the use of a validated and standardized tool to screen pregnant women at least once during the perinatal period for symptoms of depression and anxiety [47]. However, the use of multiple questionnaires to assess maternal mental health and sleep quality can be challenging in daily clinical practice, especially in an environment with a high healthcare workload. Therefore, we believe that understanding the associated risk factors may help to target those patients at higher risk and thus facilitate daily clinical practice as they can be identified at the beginning of pregnancy. Various risk factors for antenatal negative mood states have been postulated in the previous literature [29,31,32,33,34].

In our cohort, we found that a main risk factor for maternal perceived stress, a higher level of state anxiety, and poorer well-being in the third trimester was non-university studies. In line with these results, some previous research in the pregnant population had already postulated a low educational profile as a risk factor for antenatal depression [31,32]. However, in contrast to our findings, Lancaster et al. described only a small association of lower educational levels with depressive symptoms in a systematic review [33]. In general, among the non-pregnant population, a low educational level has also been associated with anxiety and depression [48]. Our results could be explained by the fact that, as previously suggested in the literature, normally, education is likely to result in good mental health rather than come from good mental health and, in turn, education may also provide success in pursuing personal ends that include emotional well-being [48,49].

For the STAI-T personality questionnaire, we found preeclampsia in a previous pregnancy to be a potential risk factor. In a systematic review, Grigoriadis et al. found that prenatal maternal anxiety was not significantly associated with preeclampsia, although there was a significant heterogeneity across studies [50]. However, we did not find any data regarding the association between previous preeclampsia and compromised mental health in subsequent pregnancies in the previous literature. A prior history of adverse obstetric events has already been related to the symptoms of anxiety and depression [31,32,51], which could be in line with our results regarding the occurrence of preeclampsia in a previous pregnancy. 

Perceived stress was also influenced in our cohort by non-white ethnicity and a maternal age of <40 years, and the latter was also found to be a risk factor for a higher score in trait anxiety among our participants. The literature also provides inconsistent findings as far as maternal age and ethnicity are concerned, as reported in the systematic reviews by Lancaster et al. [33] and Biaggi et al. [32]. In their review, Biaggi et al. described 13 studies where young age was posited as a risk factor, in contrast with 10 studies where advanced maternal age was described as a risk factor for antenatal depression and anxiety [32].

A higher level of anxiety in the third trimester and poorer maternal well-being in the third-trimester assessment were provided by the presence of a previous psychiatric disorder. These results are in line with previously published evidence, as previous mental health disorders have been strongly related to higher anxiety in the past, in particular a history of anxiety and depression and a history of psychiatric treatment [32]. Lancaster et al. also reported an association between a personal history of depression and an increased risk of antepartum depressive symptoms [33]. Multiple studies conducted during the COVID-19 pandemic on maternal mental status proposed the presence of a previous psychiatric disorder as a risk factor for negative maternal affective states [52,53,54,55].

As for poor maternal sleep quality, no significant contributing factors were found. These findings are in contrast with those found in previous research where some risk factors could be postulated as contributors to sleep disturbances during pregnancy, such as a history of stillbirth, general health-related quality of life, or insufficient physical activity [29]. Christian et al. found that African-Americans’ ethnicity and multiparity were related to poor sleep during pregnancy [56]. Other studies reported gestational age [30] or previous maternal BMI to be contributing factors [57]. Our univariate analysis also suggested ethnicity and obesity to be contributing factors; however, we could not demonstrate it in the multivariate analysis.

Finally, previous research has a well-documented association between anxiety, life stress, sleep quality, and maternal mental well-being [29,32,33]. Our results are in line with previous evidence as we found a correlation between anxiety, stress, and poorer mental well-being. In contrast, we found a low correlation between sleep quality and stress, anxiety, and mental well-being. 

On the other hand, despite these associations, we believe the use of four validated questionnaires assessing different dimensions of maternal mental health may provide a more integrative approach to overall mental health, as the absence of problems in one dimension does not necessarily guarantee the same results in other aspects of mental health.

The strengths of this study were the use of various validated questionnaires with potential clinical applicability to assess different aspects of mental health: mental stress, anxiety, well-being, and sleep quality; and that they were assessed in the second and third trimester of pregnancy, which allowed an analysis of the experimented changes throughout pregnancy. 

Among the study’s limitations is the fact that our population was a high socioeconomic cohort, with a high education profile, and most of the participants were between 30 and 40 years of age, with a low level of ethnical variety and a low proportion of obesity and gestational diabetes. This might explain some of the findings, especially in sleep disturbances, where we could not demonstrate the contribution of these factors in multivariate analysis.

We have no data regarding the first trimester of pregnancy nor the influence that these negative affective states had on perinatal results. Moreover, the neurocognitive function was not assessed, despite its potential influence on mental health [58]. In interpreting the results, it is important to understand that the use of self-reporting instruments may potentially overestimate prevalence, but it is also important to state that they also have high clinical applicability in public health and daily obstetric-care practice. Our study confirms the importance of promoting good mental health [59], especially during pregnancy.

## 5. Conclusions

Maternal stress and anxiety compromised maternal well-being, and sleep quality disturbances are very frequent and not static throughout pregnancy. Screening for these conditions at different stages of pregnancy should be recommended to professionals providing obstetric care. However, in high-pressure healthcare conditions, universal screening could be challenging; therefore, knowing the risk factors associated with these conditions can help clinicians identify pregnant women at potential risk.

## Figures and Tables

**Table 1:** Baseline characteristics of participants included in the study (n = 630).

Characteristics	Total Cohort
	n = 630
Age at recruitment (years)	35.8 (32.2–38.7)
Ethnicity	
White	497 (79.3%)
Latin	98 (15.6%)
Afro-American	6 (1%)
Asian	16 (2.6%)
Others	10 (1.6%)
Low socioeconomic status ^(a)^	25 (4%)
Study class	
Primary	25 (4%)
Secondary	176 (28%)
University	427 (68%)
BMI before pregnancy (Kg/m^2^)	23.4 (4.1)
Medical history	
Autoimmune disease	64 (10.2%)
Obesity (BMI ≥ 30)	49 (7.8%)
Thyroid disorders	35 (5.6%)
Chronic hypertension	18 (2.9%)
Psychiatric disorders ^(b)^	17 (2.7%)
Diabetes mellitus	14 (2.2%)
Chronic kidney disease	8 (1.3%)
Obstetric history	
Nulliparous	393 (62.4%)
Previous preeclampsia	17 (2.7%)
Previous stillbirth	5 (0.8%)
Use of assisted reproductive technologies	121 (19.2%)
Cigarette smoking during pregnancy	81 (12.9%)
Alcohol intake during pregnancy	14 (2.2%)
Drug consumption during pregnancy	15 (2.4%)
Sports practice during pregnancy	103 (16.3%)
Yoga or Pilates during pregnancy	141 (22.4%)

BMI: body-mass index. Data are expressed as median (IQR) or mean (SD) or n (%). ^a^ Low socioeconomic status: low (never worked or unemployed >2 years). ^b^ Psychiatric disorders: requiring therapy for psychiatric disorder.

**Table 2:** Stress, anxiety, and sleep quality of women included in the study at second and third trimester of pregnancy (n = 630).

Characteristics	2nd Trimester	3rd Trimester	*p* Value
	n = 630	n = 630	
Perceived stress scale score	16 (11–22)	16 (12–23)	0.07
State-trait Anxiety Inventory (anxiety)	13 (8–18)	14 (9–20)	<0.001
State-trait Anxiety Inventory (personality)	14 (10–21)	15 (10–21)	0.88
Five well-being Index	68 (56–76)	64 (52–76)	0.81
Pittsburg quality sleep index	7 (5–8.5)	8 (9–10)	<0.001

Data are expressed as median (IQR).

**Table 3:** Univariate and multivariate analysis of factors associated with a poor maternal PSS-14 questionnaire.

Characteristics	Univariate Analysis		Multivariate Analysis		
	OR (95% CI)	*p* Value	OR (95% CI)	*p* Value	Beta Coefficient
Maternal age < 40 years	2.24 (1.21–4.15)	0.01	2.01 (1.08–3.81)	0.03	0.705
Ethnicity					
White	0.42 (0.26–0.69)	<0.001			
Non-white	2.36 (1.45–3.83)	<0.001	2.19 (1.19–4.02)	0.01	0.786
Low socioeconomic status ^(a)^	1.82 (0.75–4.41)	0.18			
Study class					
Primary or secondary	2.19 (1.43–3.39)	<0.001	1.86 (1.08–3.19)	0.02	0.620
University	0.46 (0.30–0.70)	<0.001			
Medical history					
Obesity (BMI ≥ 30)	1.27 (0.633–2.56)	0.5			
Diabetes mellitus	1.34 (0.41–4.35)	1.34			
Autoimmune disease	1.15 (0.63–2.12)	0.65			
Thyroid disorders	0.53 (0.20–1.41)	0.2			
Psychiatric disorders ^(b)^	1.85 (0.67–5.13)	0.233			
Chronic hypertension	0.66 (0.19–2.3)	0.51			
Obstetric history					
Nulliparous	1.11 (0.73–1.71)	0.62			
Previous preeclampsia	3 (1.17–8.22)	0.02	2.7 (0.98–7.46)	0.06	0.993
Use of assisted reproductive technologies	0.58 (0.34–1.01)	0.06			
Cigarette smoking during pregnancy	0.93 (0.52–1.67)	0.81			
Alcohol intake during pregnancy	1.18 (0.74–1.87)	0.5			
Drug consumption during pregnancy	1.89 (0.62–5.75)	0.26			
Yoga or Pilates during pregnancy	0.73 (0.43–1.23)	0.24			
Constant	−2.204				
Data are expressed as n (%)					

PSS: Perceived Stress Scale; OR: Odds Ratio; CI: confidence interval; BMI: body-mass index. ^a^ Low socioeconomic status: low (never worked or unemployed >2 years). ^b^ Psychiatric disorders: requiring therapy for psychiatric disorder.

**Table 4:** Univariate and multivariate analysis of factors associated with a poor maternal STAI anxiety questionnaire.

Characteristics	Univariate Analysis		Multivariate Analysis		
	OR (95% CI)	*p* Value	OR (95% CI)	*p* Value	Beta Coefficient
Maternal age < 40 years	1.69 (0.92–3.12)	0.09			
Ethnicity					
White	0.7 (0.44–1.1)	0.12			
Non-white	1.43 (0.91–2.26)	0.12			
Low socioeconomic status ^(a)^	0.76 (0.26–2.27)	0.63			
Study class					
Primary or secondary	1.75 (1.17–2.61)	0.01	1.70 (1.11–2.59)	0.01	0.529
University	0.57 (0.38–0.85)	0.01			
Medical history					
Obesity (BMI ≥ 30)	1.01 (0.49–2.09)	0.97			
Diabetes mellitus	1.05 (0.29–3.8)	0.95			
Autoimmune disease	1.81 (1.02–3.23)	0.04	1.56 (0.84–2.89)	0.16	0.446
Thyroid disorders	2.1 (1.02–4.34)	0.04	1.81 (0.84–3.90)	0.13	0.595
Psychiatric disorders ^(b)^	3.56 (1.35–9.42)	0.01	3.62 (1.34–9.78)	0.01	1288
Chronic hypertension	0.76 (0.22–2.67)	0.67			
Chronic kidney disease	1.28 (0.25–6.42)	0.76			
Obstetric history					
Nulliparous	1.43 (0.96–2.12)	0.07			
Previous preeclampsia	1.77 (0.6–5.19)	0.3			
Previous stillbirth	0.96 (0.11–8.63)	0.97			
Use of assisted reproductive technologies	0.64 (0.37–1.01)	0.11			
Cigarette smoking during pregnancy	1.13 (0.64–2)	0.67			
Alcohol intake during pregnancy	0.92 (0.59–1.43)	0.72			
Drugs consumption during pregnancy	0.96 (0.27–3.47)	0.96			
Yoga or Pilates during pregnancy	0.5 (0.29–0.85)	0.01	0.62 (0.35–1.1)	0.1	−1.059
Constant	−1.586				
Data are expressed as n (%)					

STAI: State-Trait Anxiety Inventory; OR: Odds Ratio; CI: confidence interval; BMI: Body-mass index. ^a^ Low socioeconomic status: low (never worked or unemployed >2 years). ^b^ Psychiatric disorders: requiring therapy for psychiatric disorder.

**Table 5:** Univariate and multivariate analysis of factors associated with a poor maternal STAI personality questionnaire.

Characteristics	Univariate Analysis		Multivariate Analysis		
	OR (95% CI)	*p* Value	OR (95% CI)	*p* Value	Beta Coefficient
Maternal age < 40 years	2.28 (1.23–4.23)	0.01	2.07 (1.11–3.88)	0.02	0.729
Ethnicity					
White	0.54 (0.33–9.86)	0.02			
Non-white	1.85 (1.13–3.03)	0.02	1.55 (0.83–2.92)	0.17	−0.440
Low socioeconomic status ^(a)^	1.22 (0.47–3.19)	0.68			
Study class					
Primary or secondary	2.09 (1.36–3.22)	0.01	1.42 (0.81–2.49)	0.22	0.350
University	0.48 (0.31–7.36)	0.01			
Medical history					
Obesity (BMI ≥ 30)	0.95 (0.45–1.98)	0.88			
Diabetes mellitus	1.83 (0.60–5.58)	0.28			
Autoimmune disease	1.26 (0.69–2.3)	0.45			
Thyroid disorders	0.96 (0.42–2.16)	0.91			
Psychiatric disorders ^(b)^	2.34 (0.87–6.30)	0.09			
Chronic hypertension	0.64 (0.18–2.24)	0.48			
Chronic kidney disease	0.46 (0.05–3.75)	0.47			
Obstetric history					
Nulliparous	1.16 (0.76–1.77)	0.50			
Previous preeclampsia	3.4 (1.25–9.27)	0.01	2.9 (1.03–8.2)	0.04	1.069
Previous stillbirth	0.81 (0.09–7.29)	0.85			
Use of assisted reproductive technologies	0.79 (0.47–1.33)	0.37			
Cigarette smoking during pregnancy	1.62 (0.95–2.77)	0.08			
Alcohol intake during pregnancy	0.97 (0.6–1.55)	0.89			
Drugs consumption during pregnancy	1.3 (0.4–4.24)	0.66			
Yoga or Pilates during pregnancy	0.53 (0.3–0.92)	0.03	0.92 (0.46–1.84)	0.82	−0.079
Constant	−1.976				
Data are expressed as n (%)					

STAI: State-Trait Anxiety Inventory; OR: Odds Ratio; CI: confidence interval; BMI: body-mass index. ^a^ Low socioeconomic status: low (never worked or unemployed >2 years). ^b^ Psychiatric disorders: requiring therapy for psychiatric disorder.

**Table 6:** Univariate and multivariate analysis of factors associated with poor maternal WHO-5 questionnaire.

Characteristics	Univariate Analysis		Multivariate Analysis		
	OR (95% CI)	*p* Value	OR (95% CI)	*p* Value	Beta Coefficient
Maternal age < 40 years	1.64 (0.95–2.84)	0.07			
Ethnicity					
White	0.76 (0.46–1.25)	0.28			
Non-white	1.31 (0.80–2.15)	0.28			
Low socioeconomic status ^(a)^	1.51 (0.62–3.64)	0.36			
Study class					
Primary or secondary	1.86 (1.23–282)	0.01	1.74 (1.10–2.74)	0.02	0.553
University	0.54 (0.35–0.82)	0.01			
Medical history					
Obesity (BMI ≥ 30)	2.01 (1.07–3.79)	0.03	1.71 (0.88–3.32)	0.11	0.536
Diabetes mellitus	2.13 (0.72–6.26)	0.17			
Autoimmune disease	1.42 (0.81–2.50)	0.22			
Thyroid disorders	1.29 (0.61–2.72)	0.49			
Psychiatric disorders ^(b)^	3.27 (1.24–8.67)	0.02	2.96 (1.07–8.25)	0.04	1.087
Chronic hypertension	2.29 (0.89–5.95)	0.09			
Chronic kidney disease	0.39 (0.05–3.21)	0.38			
Obstetric history					
Nulliparous	1.49 (0.99–2.23)	0.05	1.26 (0.81–1.94)	0.29	0.231
Previous preeclampsia	1.54 (0.56–4.24)	0.41			
Previous stillbirth	0.69 (0.08–6.23)	0.74			
Assisted reproductive technologies	0.95 (0.58–1.54)	0.82			
Cigarette smoking during pregnancy	1.55 (0.92–2.62)	0.10			
Alcohol intake during pregnancy	1.24 (0.79–1.93)	0.34			
Drug consumption during pregnancy	1.56 (0.51–4.74)	0.43			
Yoga or Pilates during pregnancy	0.54 (0.32–0.91)	0.02	0.66 (0.37–1.17)	0.16	−0.413
Constant	−1.339				
Data are expressed as n (%)					

WHO-5: World Health Organization Well-Being Index Questionnaire; OR: Odds Ratio; CI: confidence interval; BMI: body-mass index ^a^ Low socioeconomic status: low (never worked or unemployed >2 years). ^b^ Psychiatric disorders: requiring therapy for psychiatric disorder.

**Table 7:** Univariate and multivariate analysis of factors associated with poor maternal Pittsburg questionnaire.

Characteristics	Univariate Analysis		Multivariate Analysis		
	OR (95% CI)	*p* Value	OR (95% CI)	*p* Value	Beta Coefficient
Maternal age < 40 years	1.08 (0.56–2.09)	0.81			
Ethnicity					
White	0.36 (0.15–0.88)	0.03			
Non-white	2.74 (1.13–6.61)	0.03	2.13 (0.86–5.30)	0.10	0.758
Low socioeconomic status ^(a)^	1.18 (0.33–4.2)	0.80			
Study class					
Primary or Secondary	1.96 (1.04–3.69)	0.04	1.91 (0.98–3.77)	0.06	0.649
University	0.51 (0.27–0.96)	0.04			
Medical history					
Obesity (BMI ≥ 30)	3.1 (0.73–13.6)	0.13			
Diabetes mellitus	0.93 (0.19–4.48)	0.93			
Autoimmune disease	1.42 (0.61–3.31)	0.42			
Thyroid disorders	0.64 (0.24–1.69)	0.37			
Psychiatric disorders ^(b)^	2.87 (0.37–22.43)	0.32			
Chronic hypertension	2.37 (0.3–18.9)	0.41			
Chronic kidney disease	0.93 (0.1–8.46)	0.95			
Obstetric history					
Nulliparous	1.16 (0.68–2)	0.59			
Use of assisted reproductive technologies	0.95 (0.51–1.8)	0.88			
Cigarette smoking during pregnancy	0.49 (0.25–0.95)	0.03	0.51 (0.25–1.02)	0.06	−0.676
Alcohol intake during pregnancy	0.76 (0.43–1.32)	0.33			
Drug consumption during pregnancy	1.94 (0.24–15.75)	0.54			
Yoga or Pilates during pregnancy	0.88 (0.48–1.58)	0.65			
Constant	1.183				
Data are expressed as n (%)					

OR: Odds Ratio; CI: confidence interval; BMI: body-mass index. ^a^ Low socioeconomic status: low (never worked or unemployed >2 years). ^b^ Psychiatric disorders: requiring therapy for psychiatric disorder.

## Data Availability

Data available subject to previous ethics committee agreement.
